# Factors Controlling the Diels–Alder Reactivity of Hetero‐1,3‐Butadienes

**DOI:** 10.1002/open.201800193

**Published:** 2018-11-26

**Authors:** Song Yu, Hans M. de Bruijn, Dennis Svatunek, Trevor A. Hamlin, F. Matthias Bickelhaupt

**Affiliations:** ^1^ Department of Theoretical Chemistry, Amsterdam Center for Multiscale Modeling (ACMM) Vrije Universiteit Amsterdam De Boelelaan 1083 1081 HV Amsterdam The Netherlands; ^2^ Leiden Institute of Chemistry, Gorlaeus Laboratories Leiden University P.O. Box 9502 2300 RA Leiden The Netherlands; ^3^ Institut für Angewandte Synthesechemie Technische Universität Wien (TU Wien) Getreidemarkt 9 1060 Vienna Austria; ^4^ Institute for Molecules and Materials (IMM) Radboud University Heyendaalseweg 135 6525 AJ Nijmegen The Netherlands

**Keywords:** activation strain model, density functional calculations, hetero-Diels–Alder reaction, orbital interactions, reactivity

## Abstract

We have quantum chemically explored the Diels–Alder reactivities of a systematic series of hetero‐1,3‐butadienes with ethylene by using density functional theory at the BP86/TZ2P level. Activation strain analyses provided physical insight into the factors controlling the relative cycloaddition reactivity of aza‐ and oxa‐1,3‐butadienes. We find that dienes with a terminal heteroatom, such as 2‐propen‐1‐imine (**NCCC)** or acrolein (**OCCC)**, are less reactive than the archetypal 1,3‐butadiene (**CCCC**), primarily owing to weaker orbital interactions between the more electronegative heteroatoms with ethylene. Thus, the addition of a second heteroatom at the other terminal position (**NCCN** and **OCCO**) further reduces the reactivity. However, the introduction of a nitrogen atom in the backbone (**CNCC**) leads to enhanced reactivity, owing to less Pauli repulsion resulting from polarization of the diene HOMO in **CNCC** towards the nitrogen atom and away from the terminal carbon atom. The Diels–Alder reactions of ethenyl‐diazene (**NNCC)** and 1,3‐diaza‐butadiene (**NCNC**), which contain heteroatoms at both the terminal and backbone positions, are much more reactive due to less activation strain compared to **CCCC**.

## 1 Introduction

One of the most iconic reactions in the field of organic chemistry are the Diels–Alder cycloadditions. Since Diels–Alder reactions were first described by Otto Diels and Kurt Alder in 1928,^1^ these [4+2] cycloadditions between 1,3‐dienes and unsaturated dienophiles have found broad applications in the synthesis of 6‐membered unsaturated ring‐systems. Diels–Alder reactions have played an important role in all fields of chemistry, from total synthesis^2^ to material science.^3^ While the archetypal [4+2] cycloadditions described by Diels and Alder featured alkenes and 1,3‐dienes as reactants, reactions between hetero‐dienes and hetero‐dienophiles are possible.^4^ These hetero‐Diels–Alder reactions are important synthetic methods for the formation of heterocycles.

**Figure 1:**
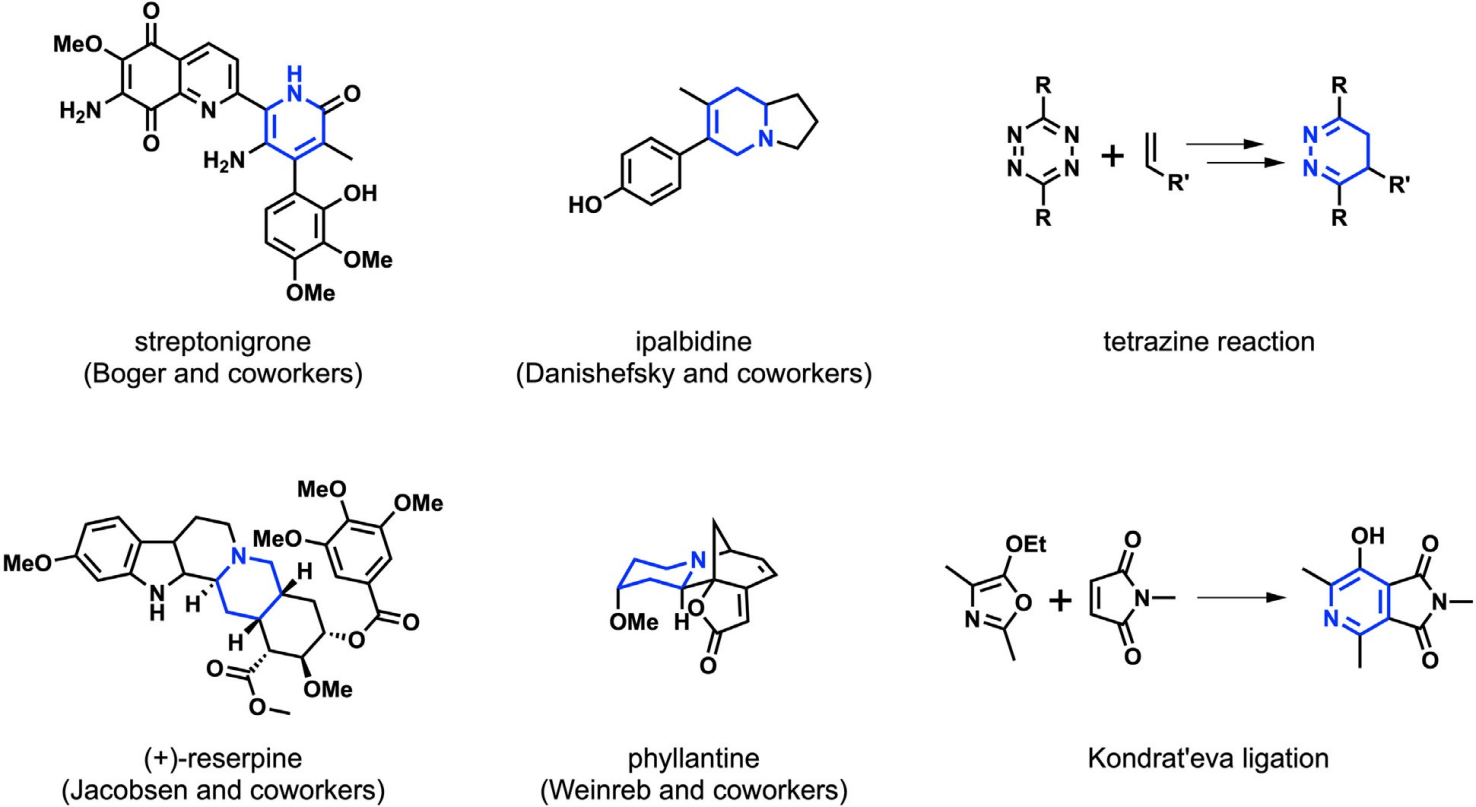
Notable natural products synthesized using an aza‐Diels–Alder cycloaddition as the key step, and two bioorthogonal ligations based on aza‐Diels–Alder reactions.

One can differentiate between different subtypes of hetero‐Diels–Alder reactions. The most common classification is based on the hetero element present in the substrates. Introduction of nitrogen into the diene or dienophile leads to aza‐Diels–Alder cycloadditions. These reactions are commonly used in total synthesis for the formation of nitrogen containing heterocyclic scaffolds. Notable examples employing aza‐Diels–Alder reactions as a key step include, among many others,^5^ the synthesis of streptonigrone by Boger and co‐workers,^6^ ipalbidine by Danishefsky and co‐workers,^7^ (+)‐reserpine by Jacobsen and co‐workers,^8^ and phyllanthine by Weinreb and co‐workers^9^ (Figure 1).

Another important aza‐Diels–Alder cycloaddition is the reaction between 1,2,4,5‐tetrazines and electron rich dienophiles. In 1964, Sauer reported on the reaction of tetrazines with dienophiles.^10^ This bioorthogonal cycloaddition proceeds through an inverse electron demand Diels–Alder reaction, followed by a cycloreversion under the loss of nitrogen, and was independently introduced by Fox and co‐workers^11^ and Weissleder and co‐workers^12^ in 2008. This ligation is often used in time‐critical applications,^13^ due to the exceptionally high possible second‐order rate constants of up to 3,300,000 m ^−1^ s^−1^.^14^ Due to the range of possible dienophiles, this bioorthogonal reaction can be applied in various applications. While *trans*‐cyclooctenes are used for high reactivity, cyclopropenes^15^ can be used for metabolic incorporation,^16^ due to their smaller size. Introduction of a carbamate in allylic position to the double bond of a *trans*‐cyclooctene allows for “click‐to‐release” reactions,^17^ opening up the possibility for targeted drug delivery.^18^ The use of vinylboronic acids^19^ can lead to high selectivity towards 2‐pyridyl^20^ or 2‐hydroxyphenyl^21^ substituted 1,2,4,5‐tetrazines, as recently shown by Bonger and co‐workers. Other bioorthogonal ligations based on aza‐Diels–Alder reactions include the 1,2,4‐triazine ligation introduced by Prescher and co‐workers^22^ and a variant of the Kondrat′eva reaction introduced by Jouanno et al. (Figure 1).^23^

Another subtype of hetero‐Diels–Alder reactions are oxo‐Diels–Alder cycloadditions. In these [4+2] cycloadditions, carbonyl compounds are used as dienophiles or 1,3‐dienes.^4^, ^24^ Due to the low reactivity of such reaction partners in predominantly inverse electron demand Diels–Alder reactions Lewis acid catalysis,^25^ cinchona alkaloid‐derived amine catalysis,^26^ or N‐heterocyclic carbene organocatalysis^27^ is often used. This also opens the possibility of enantioselective Diels–Alder cycloadditions forming pyran derivatives.^28^

Reactivities of aza‐ and oxo‐hetero‐Diels–Alder cycloadditions are found within a wide range, from unreactive to very highly reactive as observed in tetrazine ligation reactions^14^ or the Diels–Alder reactions of superelectrophiles, which show good yields with the quite unreactive ethylene at reasonably low pressure and at room temperature.^29^ However, while the kinetics of several examples of such hetero‐Diels–Alder reactions have been the subject of experimental and theoretical studies,^30^ to the best of our knowledge only one study on the influence of single nitrogen or oxygen atoms within the 1,3‐diene on the kinetics of Diels–Alder cycloadditions has been conducted. Houk and co‐workers have investigated the reactivity of cyclic and acyclic 1‐ and 2‐azadienes in Diels–Alder reactions with ethylene.^31^ They could show that the activation barrier height correlates very well with distortion energies at the transition state obtained from the distortion/interaction analysis (activation strain model) developed by Bickelhaupt and Houk.^32^ They also noted that the position of the transition state is shifted along the reaction coordinate for different systems. However, comparing interaction and strain energies for different systems at their respective transition state can lead to skewed conclusions, as for cycloadditions both the interaction and strain energy often increases along the reaction coordinate.^32^, ^33^ This means that for reactions following Hammond's postulate, systems with lower barriers of activation, and therefore earlier transition states, should have lowered strain energies at the transition state associated with them. Hence, these reactions often seem to be strain‐controlled, even when the interaction energy is the key causal factor.

Therefore, the activation strain analysis should be performed at either a consistent point of the reaction coordinate or, even better, along the entire reaction coordinate. This approach has been successfully used in the past to provide quantitative insight into cycloadditions such as 1,3‐dipolar cycloadditions,^34^ [3+2] cycloadditions^35^ and Diels–Alder reactions.33b, 36

**Scheme 1:**
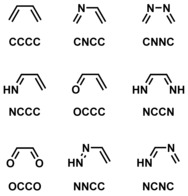
Dienes included in the present study.

We therefore aimed for an in‐depth systematic investigation on the factors controlling the reactivity of oxo‐ and aza‐hetero‐dienes (Scheme 1) in Diels–Alder cycloadditions using the activation strain model in combination with a quantitative molecular orbital (MO) theory and associated canonical energy decomposition scheme. This allows for a quantitative analysis of different factors influencing the reactivity, such as strain energy, Pauli repulsion, orbital interactions and electrostatic interactions.

## Computational Details

All calculations were carried out in ADF.2017^37^ using the BP86^38^ functional in combination with the TZ2P^39^ basis set. This exchange and correlation functional has been proven to adequately reproduce relative trends in activation energies and reaction energies for various cycloadditions.^40^ Vibrational frequency calculations were performed to verify energy minima and transition states.^41^ Local minima had zero imaginary frequencies, while transition states had a single imaginary frequency. The intrinsic reaction coordinate (IRC) method was used to follow the imaginary eigenvector towards both the reactant complex and the cycloadduct. All relative energies are with respect to the *s‐cis* conformation of the diene. Optimized structures were illustrated using CYLview.^42^

Quantitative analyses of the activation barriers associated with the studied Diels–Alder reactions are obtained by means of the activation strain model (ASM), which involves decomposing the potential energy surface Δ*E*(ζ) along the reaction coordinate ζ into the strain Δ*E*_strain_(ζ) associated with the structural deformation of the reactants from their equilibrium geometry and the interaction Δ*E*_int_(ζ) between the deformed reactants.^32^, ^43^ The Δ*E*_strain_(ζ) is determined by the rigidity of the reactants and by the extent to which they must deform in order to achieve the geometry of the transition state. The Δ*E*_int_(ζ) is usually stabilizing and is related to the electronic structure of the reactants and how they are mutually oriented over the course of the reaction [Eq. (1)]:

(1) $$\Delta E\left(\zeta \right)=\Delta E{}_{\mathrm{strain}}\left(\zeta \right)\;+\;\Delta E{}_{\mathrm{int}}\left(\zeta \right)$$

A deeper understanding of the interaction energy can be obtained using an energy decomposition analysis (EDA), in which the Δ*E*_int_(ζ) between the deformed reactants is decomposed, within the conceptual framework provided by the Kohn–Sham molecular orbital (KS‐MO) model, into three physically meaningful terms [Eq. (2)]:^44^

(2) $$\Delta E{}_{\mathrm{int}}\left(\zeta \right)=\Delta V{}_{\mathrm{elstat}}\left(\zeta \right)\;+\;\Delta E{}_{\mathrm{Pauli}}\left(\zeta \right)\;+\;\Delta E{}_{\mathrm{oi}}\left(\zeta \right)$$

The Δ*V*_elstat_(ζ) term corresponds to the classical electrostatic interaction between unperturbed charge distributions ρ_A_(*r*) + ρ_B_(*r*) of the deformed fragments A and B and is usually attractive. The Pauli repulsion Δ*E*_Pauli_(ζ) comprises the destabilizing interactions between occupied orbitals and is responsible for any steric repulsion. The orbital interaction Δ*E*_oi_(ζ) accounts for charge transfer (interaction between occupied orbitals on one fragment with unoccupied orbitals of the other fragment) and polarization (empty‐occupied orbital mixing on one fragment due to the presence of another fragment).

In activation strain diagrams and associated EDA plots in this study, the IRC is projected onto the average distance of two newly forming bonds. The resulting reaction coordinate ζ undergoes a well‐defined change in the course of the reaction from the reactant complex to the transition state and cycloadducts. The analyses along the reaction coordinate were performed with the aid of the PyFrag program.^45^

## 2 Results and Discussion

**Figure 2:**
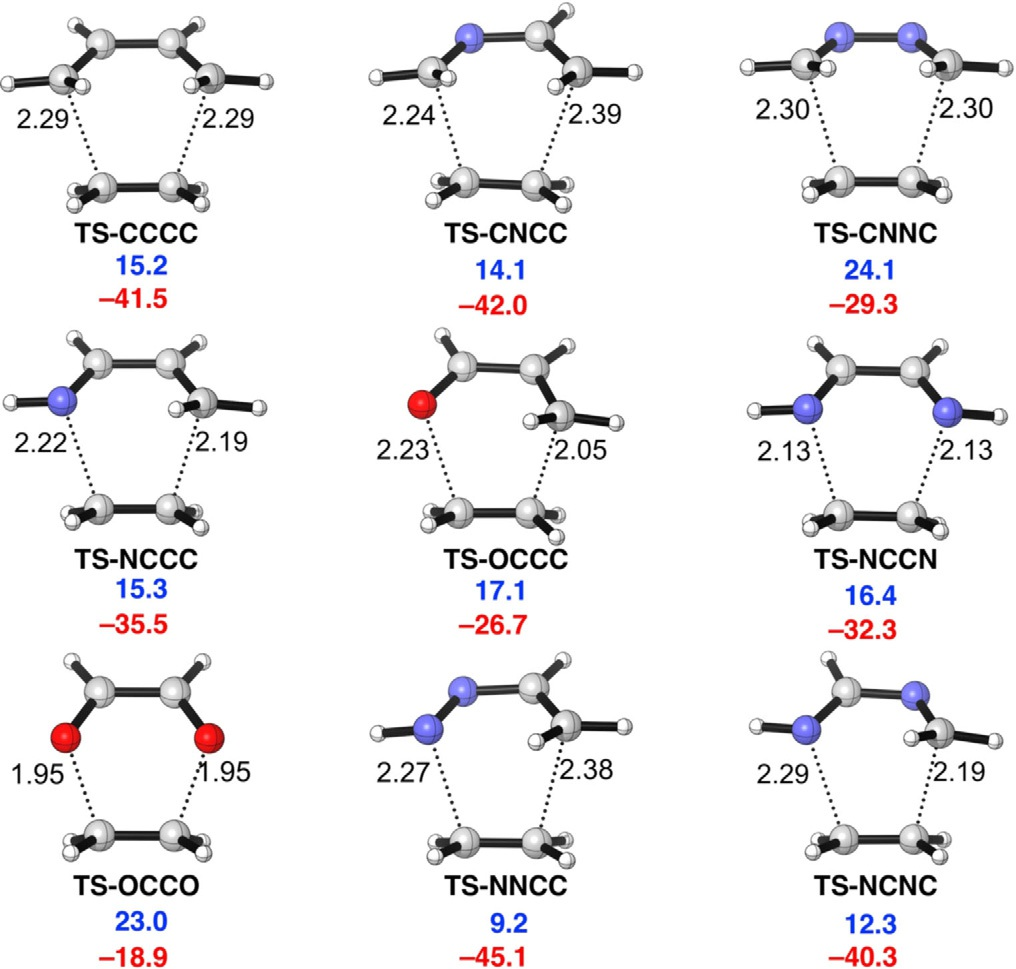
Transition structures with forming bond lengths (Å), computed activation energies (Δ*E* ^≠^, blue, kcal mol^−1^), and reaction energies (Δ*E*_rxn_, red, kcal mol^−1^) for the Diels–Alder reactions of butadiene and heterobutadienes with ethylene (**e**), computed at the BP86/TZ2P level.

Figure 2 shows transition states for Diels–Alder reactions of butadiene and hetero‐butadienes with ethylene (**e**). The computed activation energies (blue) and reaction energies (red), in kcal mol^−1^, are shown below each structure. The archetypal Diels–Alder reaction between 1,3‐butadiene (**CCCC**) with **e** has a moderate activation energy of 15.2 kcal mol^−1^. The hetero‐butadienes **CNNC**, **NCCC**, **OCCC**, **NCCN**, and **OCCO** are less reactive towards **e** compared to **CCCC**. Diels–Alder reactions of the hetero‐butadienes containing a single heteroatom in the backbone (**CNCC**, **NNCC**, **NCNC**) are more reactive than the reaction of **CCCC**, the fastest cycloaddition being with ethenyl‐diazene (**NNCC**).

The distances of forming bonds in the transition states are shown in Figure 2. Compared to the transition state **TS‐CCCC**, with the forming bond distance of 2.29 Å, **TS‐NCCC**, **TS‐OCCC**, **TS‐NCCN**, and **TS‐OCCO** have shorter average forming bonds. The shift towards later transition states is consistent with higher barrier energies and less exothermic reaction energies. **TS‐CNCC** and **TS‐NNCC** have longer average forming bonds than **TS‐CCCC**, and the reactions of **CNCC** and **NNCC** have lower barrier energies and are more exothermic than the reaction of **CCCC**. The cases outlined above are in line with the Hammond's postulate. However, **TS‐NCNC** has a shorter average bond forming distance and the reaction is less exothermic than **TS‐CCCC**, but also has a lower barrier than **TS‐CCCC. TS‐CNNC** has a longer average bond forming distance than **TS‐CCCC**, but the barrier is much higher, and the reaction is much less exothermic compared to **TS‐CCCC**. To provide a rationale for the differences in activation barriers for these Diels–Alder reactions, we undertook a combined activation strain and energy decomposition analysis study. The results are summarized below in three sections (**2.1–2.3**).

### 2.1 Diels–Alder Cycloadditions of CCCC, NCCC, NCCN, OCCC, and OCCO

**Figure 3:**
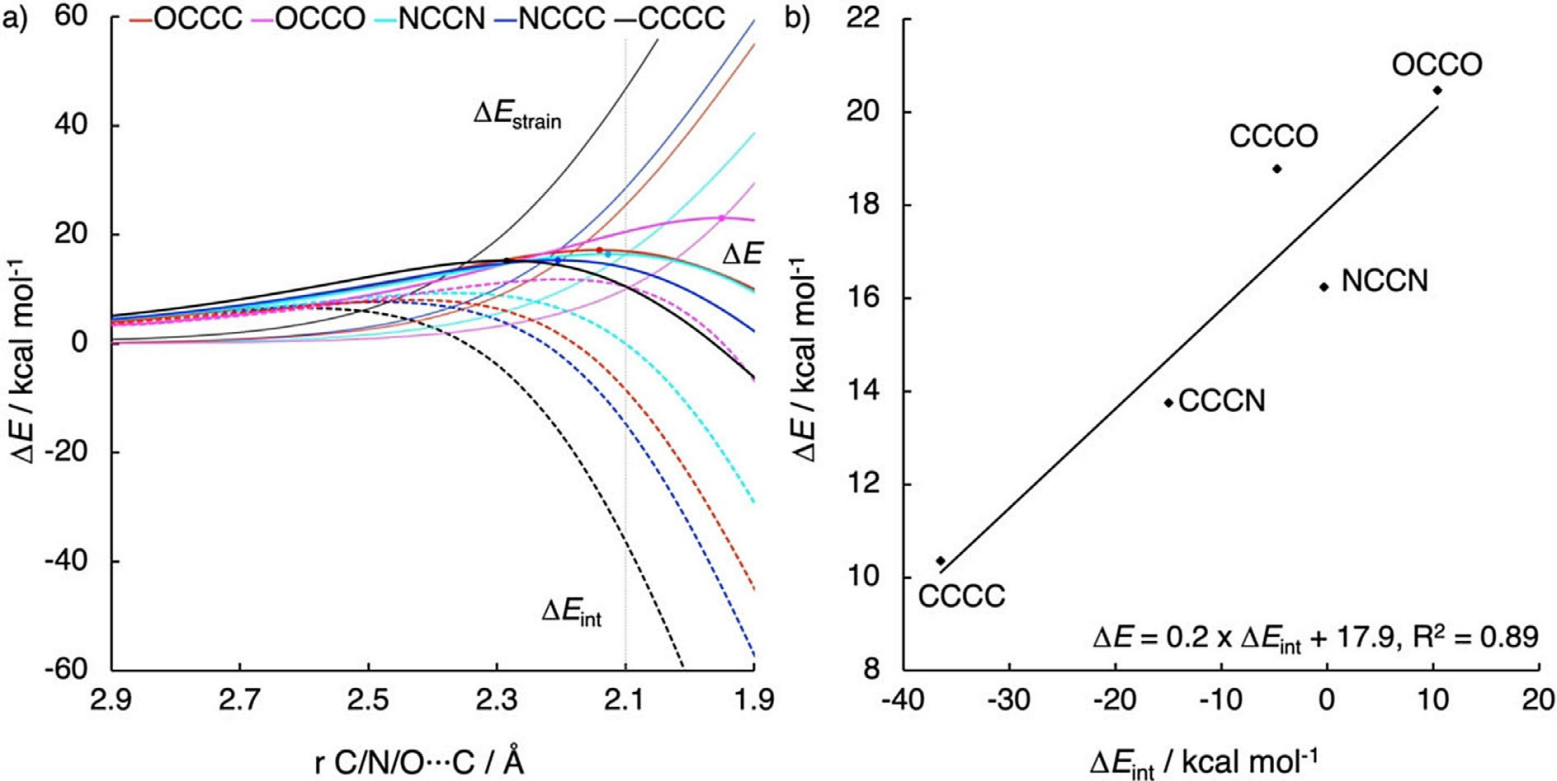
a) Activation strain analyses and b) plot of the total energies (Δ*E*) versus the interaction energies (Δ*E*_int_) of the Diels–Alder reactions between dienes **CCCC**, **NCCC**, **NCCN**, **OCCC**, and **OCCO** with ethylene (**e**) at the consistent geometry with an average C⋅⋅⋅X bond forming distance of 2.10 Å. All data were computed at the BP86/TZ2P level.

The activation strain diagram for the Diels–Alder reactions between **e** and **CCCC**, **NCCC**, **NCCN**, **OCCC**, and **OCCO** is shown in Figure 3 a. The terminal atoms of these dienes are systematically varied from carbon to nitrogen to oxygen. To be able to compare the different systems, energies will be compared at a consistent point along the reaction coordinate with an average bond forming distance of 2.10 Å, since this point is close, in both energy and position, to all TSs. **CCCC** is the most reactive diene of these five dienes. Reactivity decreases upon substitution of a terminal carbon atom with a nitrogen or oxygen atom and decreases further when both terminal carbon atoms are substituted. The differences in reactivity are mainly caused by a smaller p‐orbital of the FMOs on the terminal atoms of the dienes with increasing electronegativity of the terminal atoms.40a, 46 For this reason the oxa‐dienes are less reactive than their respective aza‐dienes.

The total energies at the consistent geometry (Figure 3 a) as well as the heights of the activation barriers of the reactions (Figure 2) of dienes **NCCC**, **NCCN**, **OCCC**, and **OCCO** are larger than those of **CCCC**. In addition, the total energies at the consistent geometry are larger for the oxa‐butadienes (**OCCC** and **OCCO**) than for the aza‐butadienes (**NCCC** and **NCCN**). We find that Δ*E*_int_ follows the trend of Δ*E*: it is more stabilizing for systems with a lower Δ*E* and a correlation is found between Δ*E* and Δ*E*_int_ at the consistent geometry (Figure 3 b). Δ*E*_strain_ increases with decreasing Δ*E*. Therefore, Δ*E*_int_ governs the differences in Δ*E* between the systems. This conclusion is consistent with our previous findings for cycloalkene Diels–Alder33b,36a and aza‐1,3‐dipolar cycloadditions.40a The differences between the systems will be further discussed by the comparison of one set of dienes (**CCCC**, **NCCC**, and **OCCC**) based on the ASM, EDA, and Frontier Molecular Orbital (FMO) analyses. Analyses of the two other sets of dienes (**CCCC**, **NCCC**, **NCCN** and **CCCC**, **OCCC**, **OCCO**) provided similar results and can be found in the Supporting Information.

**Figure 4:**
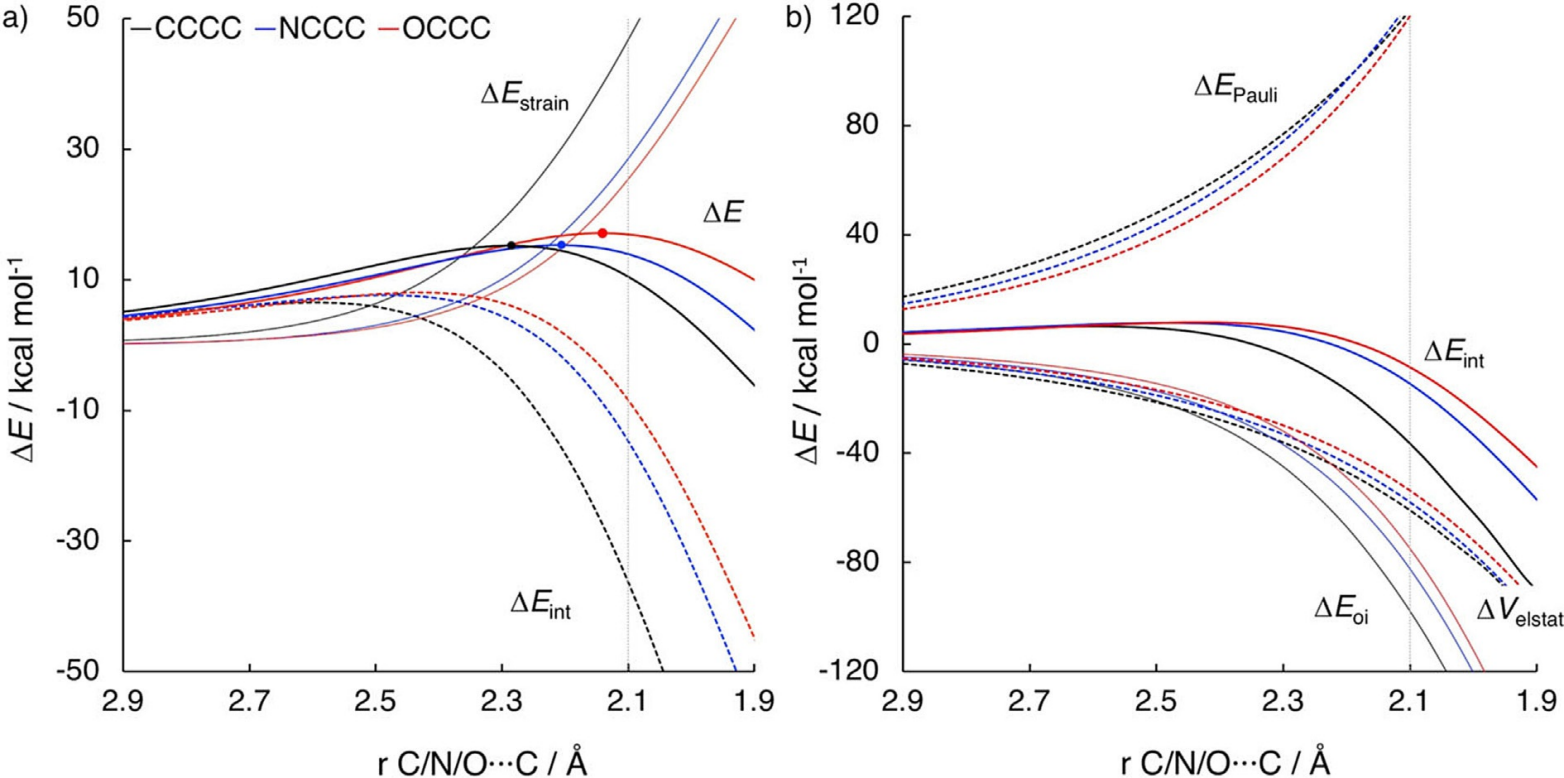
a) Activation strain analyses and b) energy decomposition analyses of the Diels–Alder reactions between dienes **CCCC**, **NCCC** and **OCCC** with ethylene (**e**) computed at the BP86/TZ2P level.

The ASM and EDA diagrams for the Diels–Alder reactions of **CCCC** (black), **NCCC** (blue), and **OCCC** (red) with **e** are shown in Figure 4 a and Figure 4 b, respectively. At the consistent geometry, Δ*E*_strain_ decreases going from **CCCC** to **NCCC** to **OCCC** due to the decreased number of terminal hydrogens in the hetero‐butadienes, which need to be bent away during the reaction. However, this decrease in Δ*E*_strain_ does not yield a lower Δ*E* for the Diels–Alder reactions of these hetero‐butadienes: Δ*E*_int_ plays a decisive role and governs the trends in Δ*E*. Decomposition of Δ*E*_int_ shows that Δ*E*_int_ is controlled by Δ*E*_oi_ and less so by Δ*V*_elstat_, while Δ*E*_Pauli_ follows a trend opposite that of Δ*E*_int_ (Figure 4 b).

**Figure 5:**
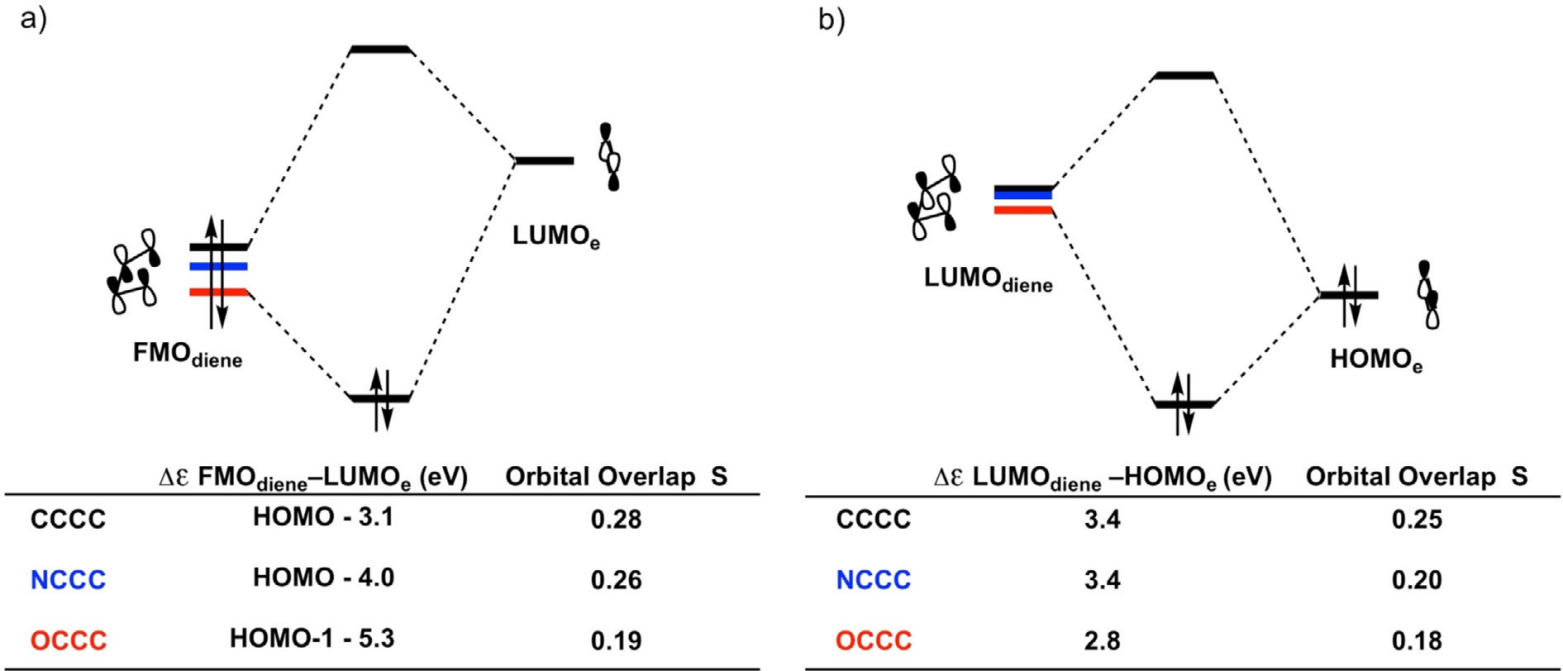
a) MO diagrams with calculated energy gaps and orbital overlaps for the normal demand FMO_diene_–LUMO_**e**_ interaction and b) the inverse demand LUMO_diene_–HOMO_**e**_ interaction of the Diels–Alder reactions between dienes **CCCC**, **NCCC**, and **OCCC** with ethylene (**e**). All data were computed at the BP86/TZ2P level at the consistent geometry with an average C⋅⋅⋅X bond forming distance of 2.10 Å.

The differences in Δ*E*_oi_ are caused by the decrease in the size of the lobe of the FMO_diene_ and LUMO_diene_ on the terminal atom, as it changes from C to N to O, due to the more compact nature of the 2p orbital of the nitrogen and oxygen atom, and by a decrease of the energy levels of both the occupied FMO and LUMO of the diene.40a, 46 The overlap and energy gaps between the FMO_diene_‐LUMO_**e**_ and the LUMO_diene_‐HOMO_**e**_ (for the normal and inverse electron demand orbital interaction, respectively) are shown in Figure 5 a and 5b. Δ*E*_oi_ is most stabilizing for **CCCC**, and becomes weaker going to **NCCC** and **OCCC**. This destabilization is reflected in the FMO_diene_‐LUMO_**e**_ and LUMO_diene_‐HOMO_**e**_ gaps and the overlap between these orbitals. For the normal electron demand orbital interaction, the orbital energy gap and the overlap between the FMO_diene_ and LUMO_**e**_ are smallest (3.1 eV) and largest (0.28) respectively for **CCCC**, while they are largest (5.3 eV) and smallest (0.19) for **OCCC**. The HOMO‐1 of **OCCC** reacts with LUMO_**e**_ instead of the HOMO, due to the fact that the HOMO has become a lone pair MO. For the inverse electron demand orbital interaction, the orbital energy gap for **CCCC** is larger than for **OCCC** (3.4 and 2.8 eV respectively), but the overlap is much larger for **CCCC** than for **OCCC** (0.25 and 0.18 respectively), thus also yielding a more stabilizing Δ*E*_oi_ in case of **CCCC**.

### 2.2 Diels–Alder Cycloadditions of CCCC, CNCC, and CNNC

Next, we investigated the Diels–Alder reactions between **CCCC**, **CNCC**, and **CNNC** with **e**. Introducing nitrogen atoms in the backbone of butadiene raises the strain energy along the reaction coordinate, yielding the highest barrier for **CNNC**. The activation energy difference between **CNCC** and **CCCC** results from the difference in Pauli repulsion energy.

**Figure 6:**
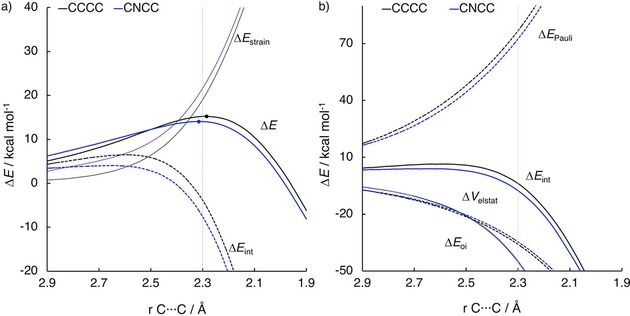
a) Activation strain analyses and b) energy decomposition analyses of the Diels–Alder reactions between dienes **CCCC** and **CNCC** with ethylene (**e**) computed at the BP86/TZ2P level.

The reaction of **CNNC** with **e** has the highest barrier due to the large deformation of the diene in the transition state with respect to the ground state. In order to react with ethylene, the dienes must adopt an *s‐cis* conformation where the dihedral angle of the backbone is <10°. For **CNNC**, the dihedral angle in the ground state is 95.6°. This has been attributed, very recently by Wiberg, Rablen, and Baraban, to the repulsion between the nitrogen lone pairs.^47^ Interestingly, the dihedral angle decreases to 55.5 and 30.7° for **CNCC** and **CCCC**, respectively. Compared to the C−C−C angle of **CCCC** (125.9°), the smaller C‐N‐C angle of **CNCC** (120.4°) leads to a larger dihedral angle of **CNCC** in order to reduce the repulsion between the terminal hydrogens on opposite ends (Figure S5).^47^ Therefore Δ*E*_strain_ is the largest for **CNNC** (which has therefore the highest barrier) and decreases with a decreasing amount of nitrogen atoms (Figure S5 and Figure 6 a). Although Δ*E*_strain_ is larger for **CNCC** than for **CCCC**, the barrier height for **CNCC** is lower, due to a more stabilizing Δ*E*_int_ along the entire reaction coordinate. The lower Δ*E*_int_ is caused by a lower Δ*E*_Pauli_, while Δ*E*_oi_ and Δ*V*_elstat_ are very similar along the reaction coordinate (Figure 6 b).

**Figure 7:**
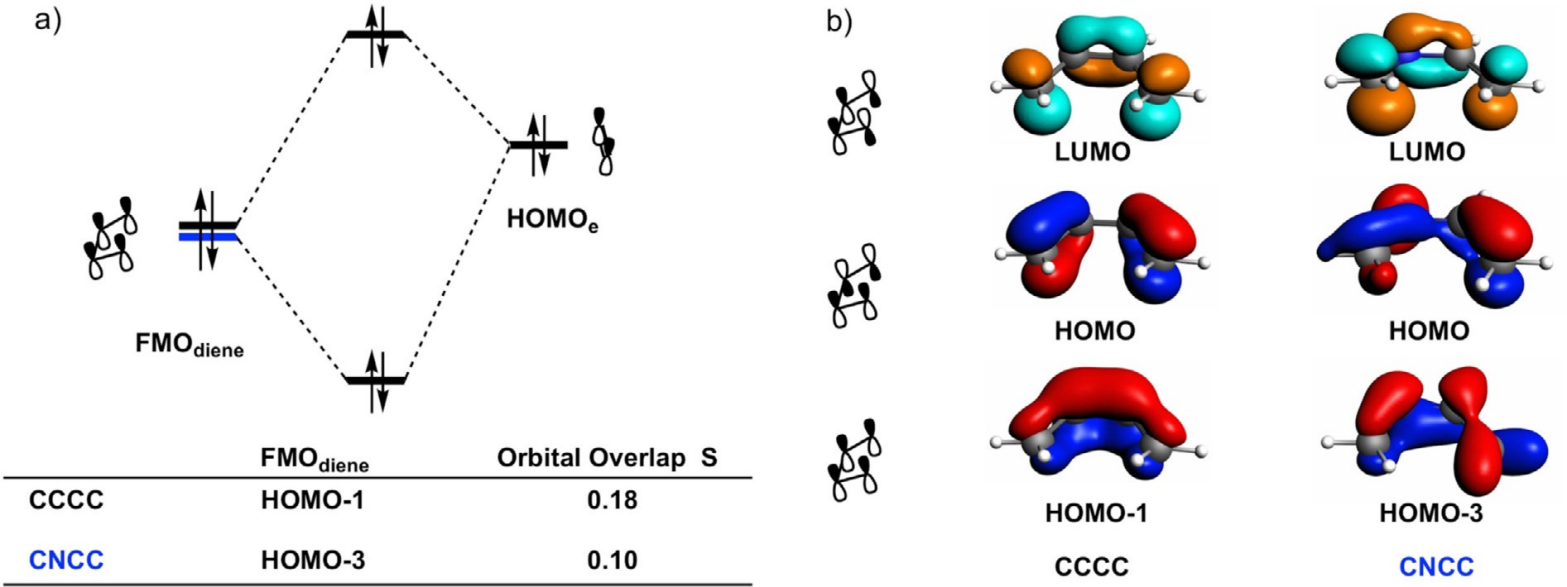
a) MO diagrams of the most significant occupied orbital overlaps of the Diels–Alder reactions between dienes **CCCC** and **CNCC** with (**e**), computed at the BP86/TZ2P level at a consistent geometry with an average C⋅⋅⋅C bond forming distance of 2.30 Å. b) FMO diagrams (isovalue=0.07) for dienes **CCCC** and **CNCC** at the consistent geometry (top row: lowest interacting virtual orbitals, middle row: highest interacting occupied orbitals, bottom row: occupied orbitals most relevant to the Pauli energies).

To rationalize the differences in the Δ*E*_Pauli_ between the Diels–Alder reactions of **CNCC** and **CCCC**, we quantified the most significant interactions between filled orbitals^48^ of the dienes and **e** (Figure 7 a) at a consistent geometry with an average C⋅⋅⋅C bond forming distance of 2.30 Å (which is close, in both energy and position, to both TSs). The highly symmetrical HOMO‐1 of **CCCC** has a large overlap with the HOMO of **e** (0.18). However, the HOMO‐3 of **CNCC** overlaps much less with the HOMO of **e** (overlap of 0.10), primarily due to the distortion of the HOMO‐3 caused by the nitrogen atom in the backbone. The decreased four electron‐two center orbital overlap for **CNCC** results in a less destabilizing Δ*E*_Pauli_ and therefore a lower activation barrier due to the more stabilizing interaction.

**Figure 8:**
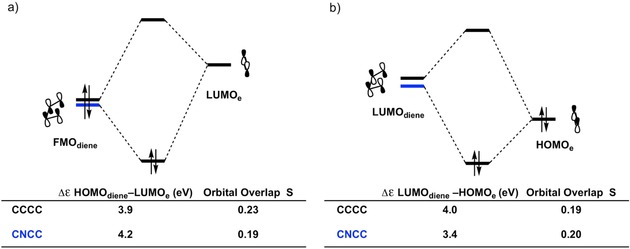
a) MO diagrams with calculated energy gaps and orbital overlaps for the normal demand FMO_diene_–LUMO_**e**_ interaction and b) the inverse demand LUMO_diene_–HOMO_**e**_ interaction of the Diels–Alder reactions between dienes **CCCC** and **CNCC** with ethylene (**e**). All data were computed at the BP86/TZ2P level at a consistent geometry with an average C⋅⋅⋅C bond forming distance of 2.30 Å.

To understand why the Δ*E*_oi_ is so similar for the reactions of **CCCC** and **CNCC**, an FMO analysis was performed (Figure 8). It turns out that the normal demand orbital interaction is more favorable for **CCCC**, while the inverse demand orbital interaction is more favorable for **CNCC**. These two interactions effectively offset each other, resulting in a very similar Δ*E*_oi_ for the two reactions. In the normal electron demand orbital interaction, the energy gap and orbital overlap between the HOMO_diene_ and LUMO_**e**_ are more favorable, i.e., smaller and larger, respectively for **CCCC** (3.9 eV and 0.23 compared to 4.2 eV and 0.19 for **CNCC**). In the inverse electron demand orbital interaction, the energy gap and orbital overlap are more favorable, i.e., smaller and larger, respectively for **CNCC** (3.4 eV and 0.20 versus 4.0 eV and 0.19 for **CCCC**), thus yielding a very similar Δ*E*_oi_. The difference in the overlap in the normal demand orbital interaction can be explained by inspecting the HOMOs of **CCCC** and **CNCC** (Figure 7 b). Compared to the HOMO of **CCCC**, the HOMO of **CNCC** has a reduced amplitude on one of the terminal carbon atoms, thus yielding a smaller overlap between the HOMO_diene_ and LUMO_**e**_. The LUMOs of both dienes are more similar on the terminal carbon atoms (Figure 7 b), resulting in a very similar overlap for the inverse demand orbital interaction.

### 2.3 Diels–Alder Cycloadditions of CNCC, NCNC, and NNCC

**Figure 9:**
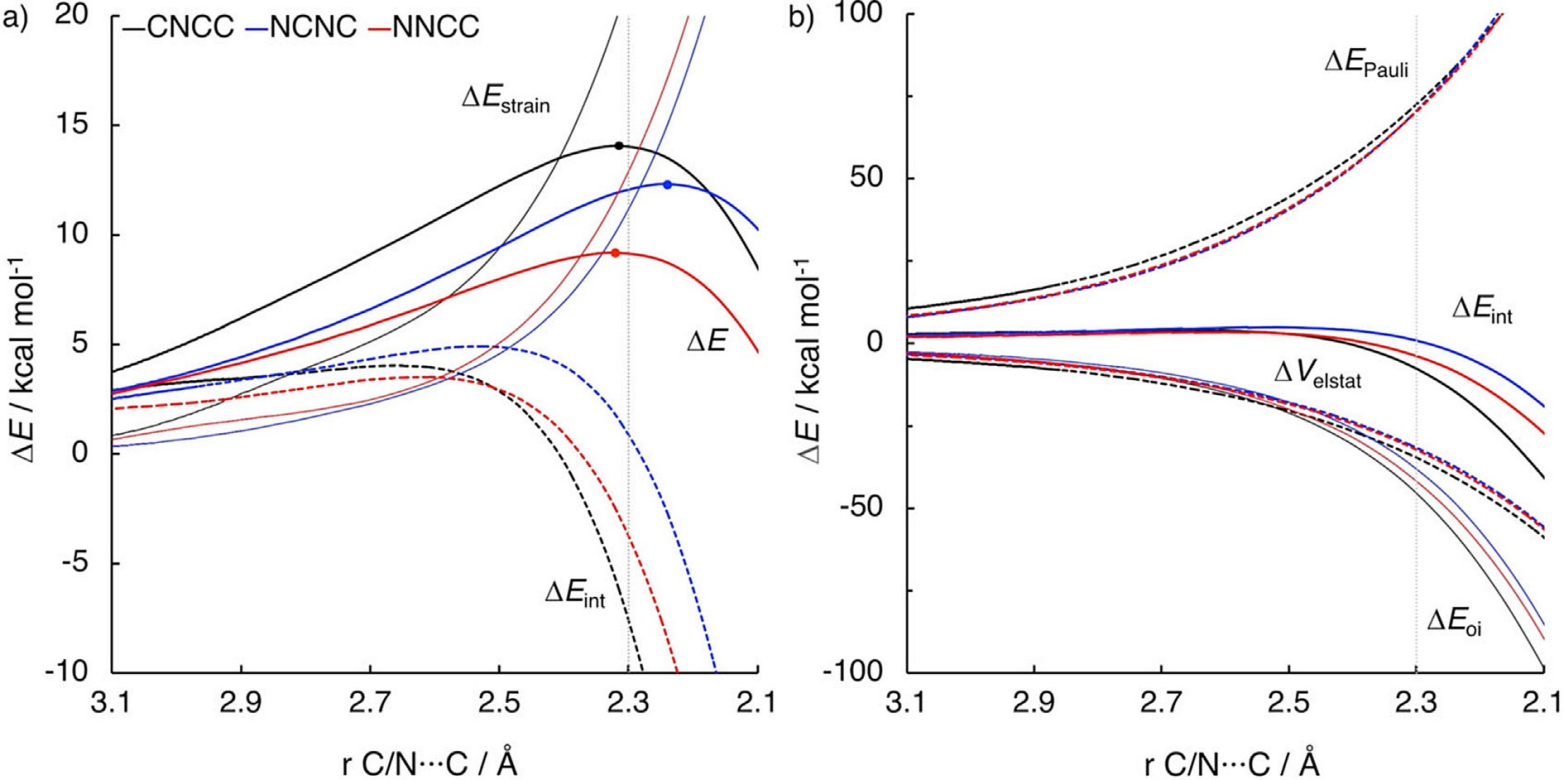
a) Activation strain analyses and b) energy decomposition analyses of the Diels–Alder reactions between dienes **CNCC**, **NCNC**, and **NNCC** with ethylene (**e**) computed at the BP86/TZ2P level.

The Diels–Alder reactions of **CNCC**, **NCNC**, and **NNCC** were compared. These dienes all contain a single nitrogen atom in the backbone, but the number and position of the nitrogen atom in the terminal sites is varied. The Diels–Alder reaction of **CNCC** with **e** has a higher barrier compared to **NCNC** and **NNCC** caused by the more destabilizing Δ*E*_strain_. This is the result of having to bend away more terminal hydrogen atoms in the case of the terminal =CH_2_ compared to =NH, as previously discussed in Section 2.1. Δ*E*_int_ is more stabilizing for **CNCC** than for both **NNCC** and **NCNC**, but is unable to compensate for the high Δ*E*_strain_ (Figure 9 a). The Diels–Alder reaction of **NNCC** has the lowest reaction barrier of the three dienes, due to the more stabilizing Δ*E*_oi_ compared to **NCNC**.

**Figure 10:**
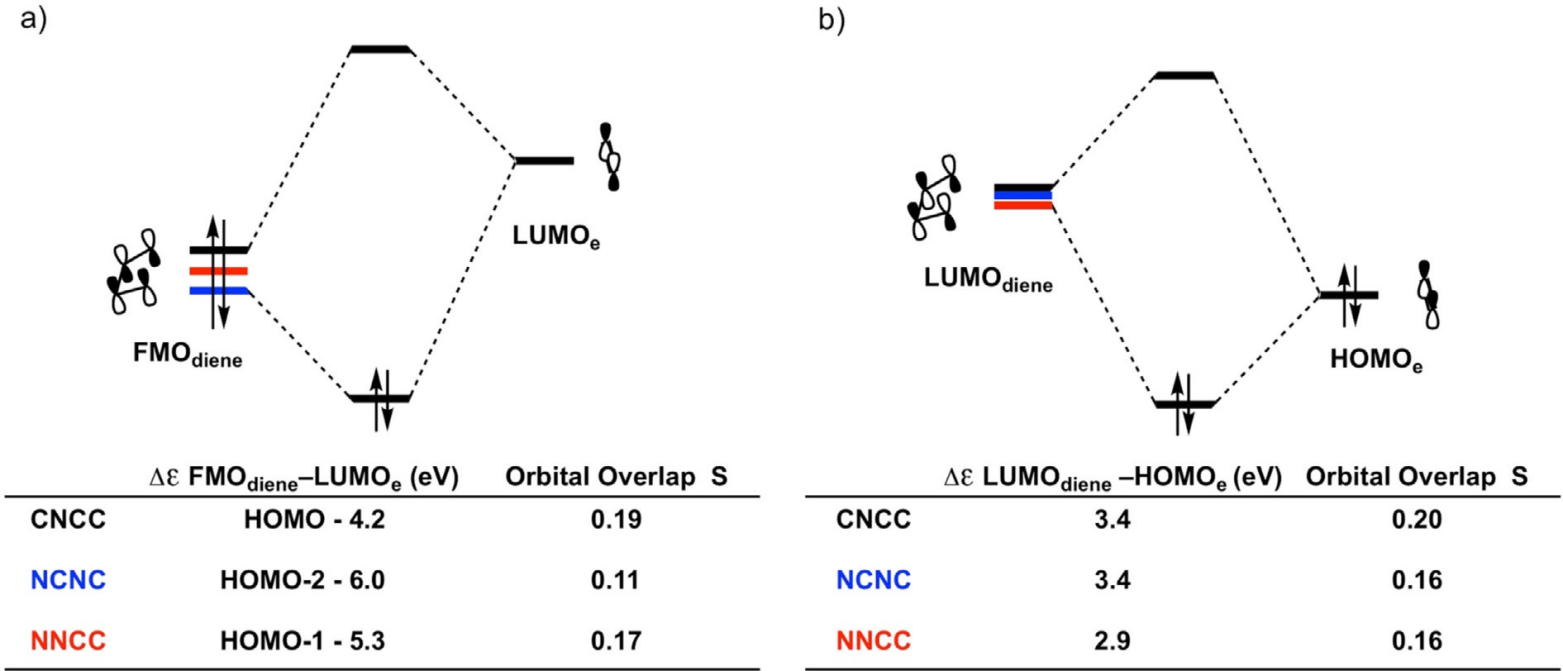
a) MO diagrams with calculated energy gaps and orbital overlaps for the normal demand FMO_diene_–LUMO_**e**_ interaction and b) the inverse demand LUMO_diene_–HOMO_**e**_ interaction of the Diels–Alder reactions between dienes **CNCC**, **NCNC**, and **NNCC** with ethylene (**e**). All data were computed at the BP86/TZ2P level at a consistent geometry with an average C⋅⋅⋅X bond forming distance of 2.30 Å.

The lower barrier for **NNCC** compared to **NCNC** is determined by Δ*E*_int_, since Δ*E*_strain_ for these reactions follows the opposite trend of the Δ*E*. The decomposition of Δ*E*_int_ (Figure 9 b) shows that Δ*E*_oi_ is the sole factor determining the height of Δ*E*_int_. To understand the difference in Δ*E*_oi_, an FMO analysis was performed for both the normal and inverse electron demand orbital interactions at a consistent geometry with an average C⋅⋅⋅X bond forming distance of 2.30 Å, which has been chosen since it is close, in both energy and position, to all TSs (Figure 10). The more stabilizing Δ*E*_oi_ for **NNCC** is due to smaller FMO gaps between the interacting orbitals in both the normal and inverse demand orbital interactions (5.3 and 2.9 eV respectively for **NNCC** compared to 6.0 and 3.4 eV for **NCNC**) and to a larger FMO_diene_‐LUMO_**e**_ overlap for **NNCC** (0.17 versus 0.11 for **NCNC**). The decreased orbital overlap for **NCNC** in the normal demand orbital interaction is due the presence of the nitrogen atom directly adjacent to the terminal carbon atom. This adjacent nitrogen atom effectively reduces the electron density on the terminal carbon atom, resulting in a smaller lobe of the FMO_diene_ on the carbon atom and a less efficient overlap between the FMO_diene_ and the LUMO_**e**_. The LUMOs of **NNCC** and **NCNC** are very similar in shape and size, resulting in the same overlap (0.16) between the LUMO_diene_ and the HOMO_**e**_ at a consistent forming bond length (see Figure S6).

## 3 Conclusions

The replacement of carbon atoms by heteroatoms in 1,3‐butadiene (**CCCC)** dramatically influences the Diels–Alder reactivity of these dienes with ethylene. Dienes with a terminal heteroatom (**NCCC** and **OCCC**) are less reactive than **CCCC** and replacement of the other terminal carbon atom by nitrogen or oxygen further decreases the reactivity. Replacing one of the carbon atoms in the backbone by nitrogen (**CNCC**) enhances the reactivity compared to **CCCC**. The replacement of two carbon atoms, one at the terminal position and one in the backbone (**NNCC** and **NCNC**), yields even more reactive systems.

For dienes in which one or two terminal carbon atoms are replaced by heteroatoms, the Diels–Alder reaction rate is decreased. The reason is the combination of a more contracted and lower energy p‐orbital on the heteroatom in the highest occupied π‐type orbital of the diene, which weakens the stabilizing donor‐acceptor orbital overlap and interaction with the ethylene LUMO. This factor dominates a counteracting influence of the activation strain, which generally decreases as the number of terminal element−H bonds that have to bend away becomes smaller. However, introduction of a nitrogen atom in the backbone (**CNCC**) furnishes a more reactive diene compared to **CCCC**, primarily due to a less destabilizing Pauli repulsion. This effect was traced back to the polarized nature of the HOMO of **CNCC** towards the nitrogen atom and away from the terminal carbon atom. Consequently, the four electron‐two center overlap between the HOMO of **CNCC** and HOMO of **e** is reduced.

The reactivity of hetero‐1,3‐butadienes with ethylene turns out to be a delicate interplay between the overlap of bond forming orbitals, the energy levels of those orbitals, and the overlap of filled orbitals on both substrates. We envision dienes containing nitrogen atoms in the backbone (2‐azadienes) to be more reactive than their all‐carbon counterparts, while addition of heteroatoms on the bond forming positions (1‐azadienes) to result in less reactive dienes, which is consistent with previous studies.^31^ However, the combination of nitrogen atoms in one of the bond forming positions and in one of the backbone positions yields the most reactive diene. We believe these insights to be valuable in the design of Diels–Alder reactions in the future.

## Conflict of interest

*The authors declare no conflict of interest*.

## Supporting information

As a service to our authors and readers, this journal provides supporting information supplied by the authors. Such materials are peer reviewed and may be re‐organized for online delivery, but are not copy‐edited or typeset. Technical support issues arising from supporting information (other than missing files) should be addressed to the authors.

Supplementary
