# Supplementary material for: Factors Controlling the Diels–Alder Reactivity of Hetero‐1,3‐Butadienes
Source: ChemistryOpen. 2018 Nov 26;7(12):995–1004. doi: 10.1002/open.201800193 (PMC6276106; doi:10.1002/open.201800193)
Supplement: Supplementary file 1 — Supplementary [file OPEN-7-995-s001.pdf]

ChemistryOPEN

Supporting Information

© 2018 The Authors. Published by Wiley-VCH Verlag GmbH & Co. KGaA, Weinheim

Factors Controlling the Diels–Alder Reactivity of Hetero-1,3-Butadienes

Song Yu+,[a] Hans M. de Bruijn+,[a, b] Dennis Svatunek,[a, c] Trevor A. Hamlin,*[a] and F. Matthias Bickelhaupt*[a, d]

open_201800193_sm_miscellaneous_information.pdf

Contents

Figure S1. (a) Activation strain analyses and (b) energy decomposition analyses of the Diels-Alder reactions between dienes **CCCC**, **OCCC**, and **OCCO** with ethylene (**e**).

Figure S2. (a) MO diagrams for the normal demand interaction and (b) the inverse demand interaction for the Diels-Alder reactions between dienes **CCCC**, **OCCC**, and **OCCO** with ethylene (**e**).

Figure S3. (a) Activation strain analyses and (b) energy decomposition analyses of the Diels-Alder reactions between dienes **CCCC**, **NCCC**, and **NCCN** with ethylene (**e**).

Figure S4. (a) MO diagrams for the normal demand interaction and (b) the inverse demand interaction for the Diels-Alder reactions between dienes **CCCC**, **NCCC**, and **NCCN** with ethylene (**e**).

Figure S5. (a) Activation strain analyses and (b) strain energy decomposition of the Diels-Alder reactions between dienes **CCCC**, **CNCC**, and **CNNC** with ethylene (**e**).

Figure S6. FMO diagrams (isovalue = 0.07) for dienes **NCNC** and **NNCC** at the consistent geometry (top row: interacting virtual orbitals, bottom row: interacting occupied orbitals).

Table S1. Cartesian coordinates, electronic energies, and imaginary vibrational frequencies.

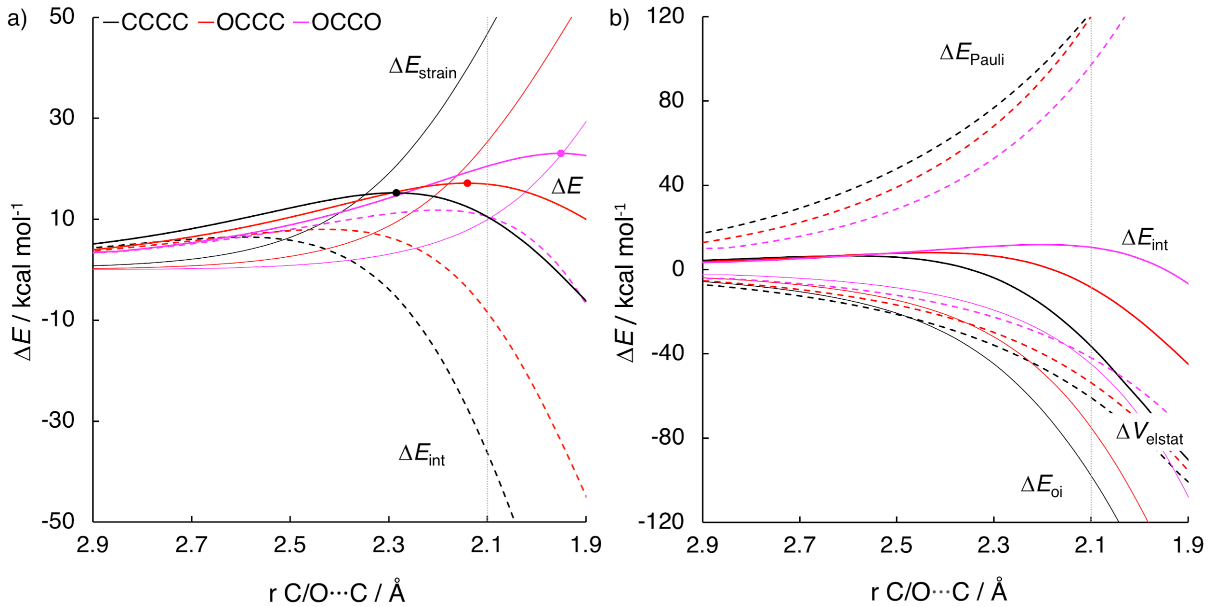

Figure S1. (a) Activation strain analyses and (b) energy decomposition analyses of the Diels-Alder reactions between dienes **CCCC**, **OCCC**, and **OCCO** with ethylene (**e**) computed at the BP86/TZ2P.

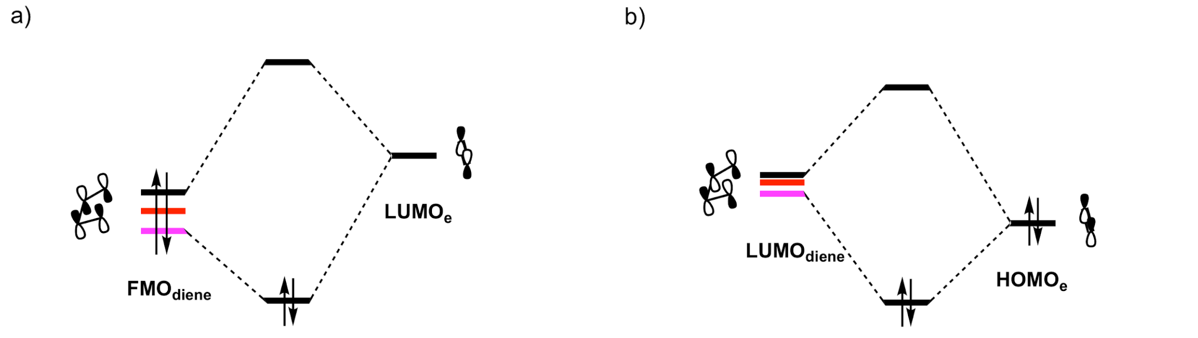

	Δε FMO_{diene}–$LUMO_e$ (eV)	Orbital Overlap S
CCCC	HOMO - 3.1	0.28
OCCC	HOMO-1 - 5.3	0.19
OCCO	HOMO-2 - 8.2	0.13

	Δε $LUMO_{diene}$ –$HOMO_e$ (eV)	Orbital Overlap S
CCCC	3.4	0.25
OCCC	2.8	0.18
OCCO	1.7	0.09

Figure S2. (a) MO diagrams with calculated energy gaps and orbital overlaps for the normal demand FMO_{diene}–$LUMO_e$ interaction and (b) the inverse demand $LUMO_{diene}$–$HOMO_e$ interaction from the Diels-Alder reactions between dienes **CCCC**, **OCCC**, and **OCCO** with ethylene (**e**). All data computed at the BP86/TZ2P level at a consistent geometry, where average C···X bond forming distances of 2.10 Å.

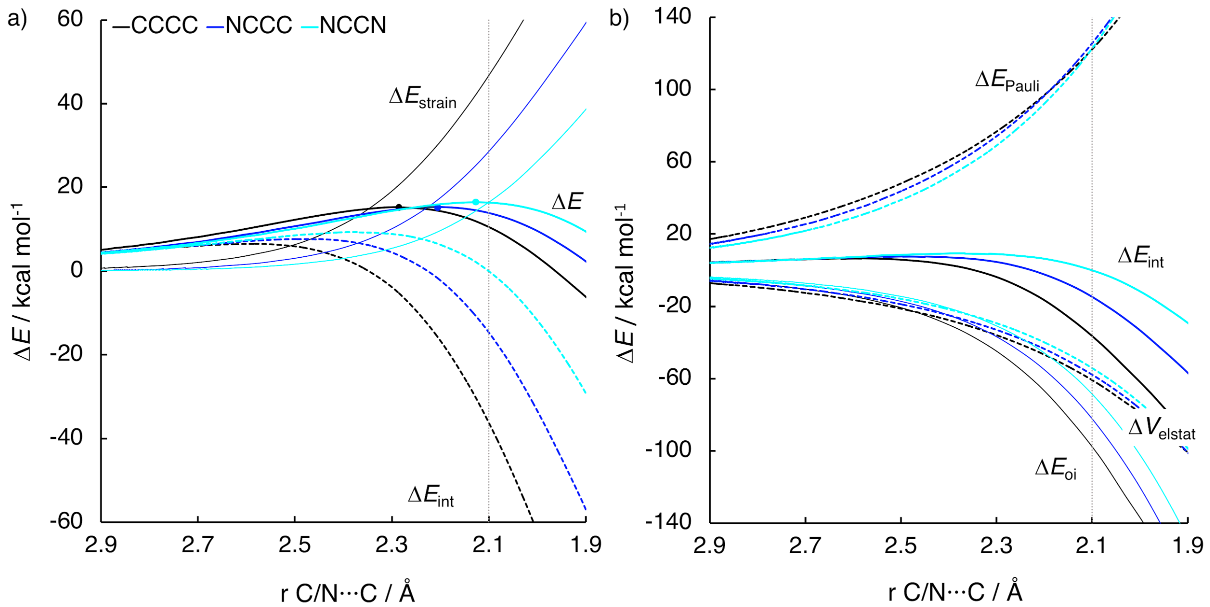

Figure S3. (a) Activation strain analyses and (b) energy decomposition analyses of the Diels-Alder reactions between dienes **CCCC**, **NCCC**, and **NCCN** with ethylene (**e**) computed at the BP86/TZ2P level.

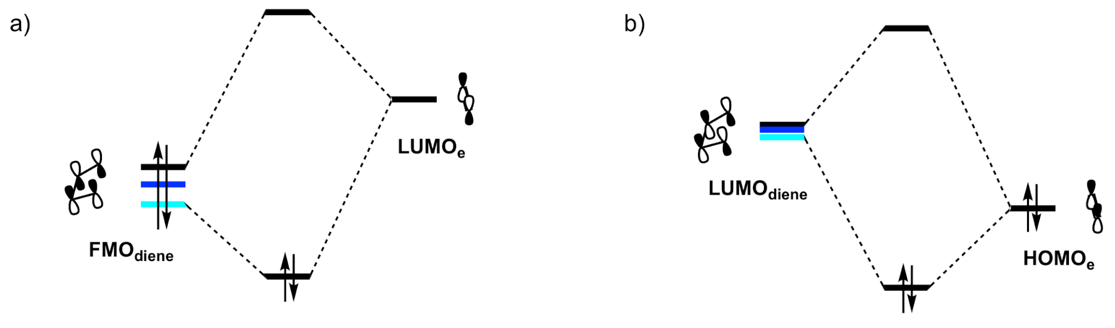

	Δε FMO_{diene}–$LUMO_e$ (eV)	Orbital Overlap S
CCCC	HOMO - 3.1	0.28
NCCC	HOMO - 4.0	0.26
NCCN	HOMO-2 - 5.5	0.13

	Δε $LUMO_{diene}$–$HOMO_e$ (eV)	Orbital Overlap S
CCCC	LUMO - 3.4	0.25
NCCC	LUMO - 3.4	0.20
NCCN	LUMO - 3.1	0.14

Figure S4. (a) MO diagrams with calculated energy gaps and orbital overlaps for the normal demand FMO_{diene}–$LUMO_e$ interaction and (b) the inverse demand $LUMO_{diene}$–$HOMO_e$ interaction from the Diels-Alder reactions between dienes **CCCC**, **NCCC**, and **NCCN** with ethylene (**e**). All data computed at the BP86/TZ2P level at a consistent geometry, where average C···X bond forming distances of 2.10 Å.

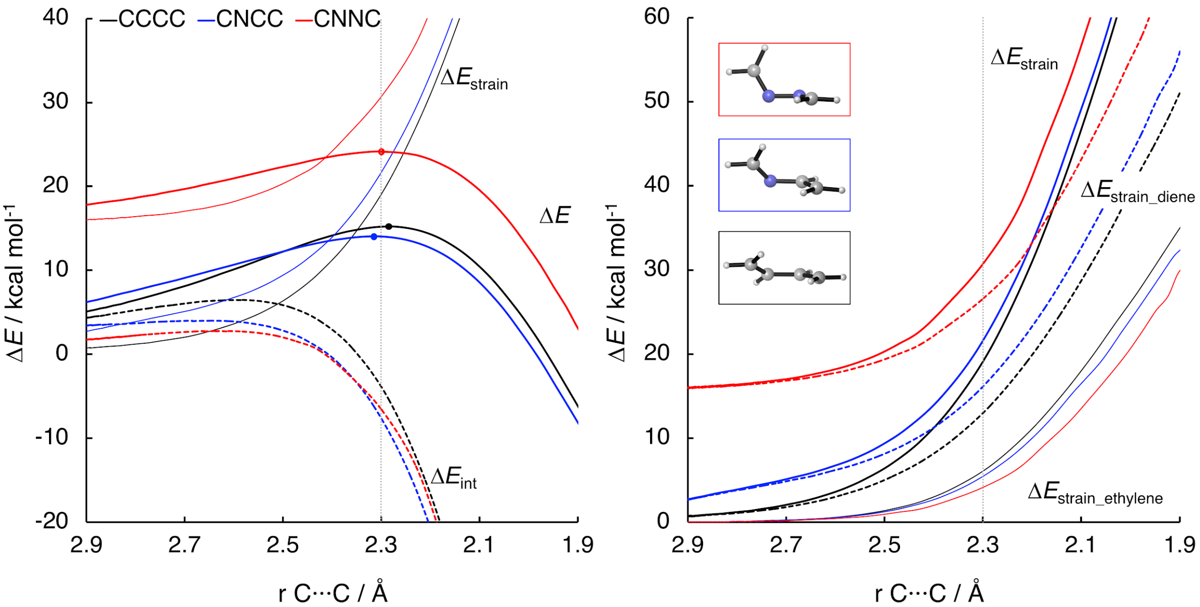

Figure S5. (a) Activation strain analyses and (b) strain energy decomposition of the Diels-Alder reactions between dienes **CCCC**, **CNCC**, and **CNNC** with ethylene (**e**) computed at the BP86/TZ2P level. The structures in boxes illustrate that the dihedral angle of the backbone in ground states decrease from **CNNC** to **CNCC** to **CCCC**.

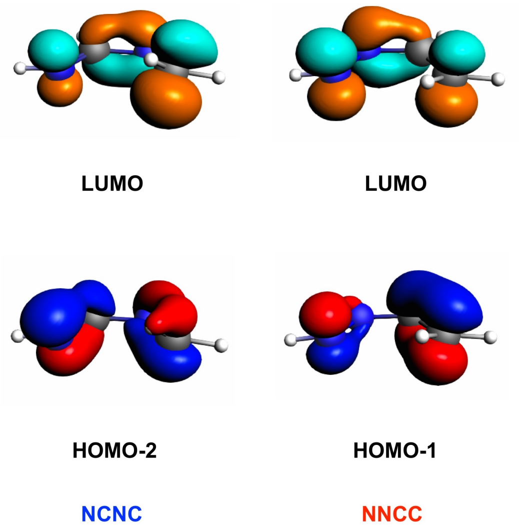

Figure S6. FMO diagrams (isovalue = 0.07) for dienes **NCNC** and **NNCC** at the consistent geometry (top row: interacting virtual orbitals, bottom row: interacting occupied orbitals).

Table S1. Cartesian coordinates (Å), energies (kcal mol^{-1}), and imaginary vibrational frequencies of the optimized structures.

CCCC - butadiene
E = −1294.63
N_{imag} = 0

C	-1.468527	-0.484641	1.674110
C	-0.726457	-0.096429	2.721232
C	0.726457	0.096429	2.721232
C	1.468527	0.484641	1.674110
H	-2.549424	-0.589357	1.755554
H	-1.017564	-0.725101	0.710558
H	-1.226404	0.071735	3.679826
H	1.226404	-0.071735	3.679826
H	1.017564	0.725101	0.710558
H	2.549424	0.589357	1.755554

TS-CCCC
E = −2008.54
N_{imag} = 1, ν = −441.030i cm^{-1}

C	0.810099	0.702461	-0.341801
C	2.645320	2.913587	0.088943
C	0.678660	1.813935	0.469122
C	1.878380	-0.204138	-0.202961
C	2.862720	-0.040256	0.753251
C	3.696497	2.022919	0.228348
H	0.195798	0.633908	-1.242208
H	2.413163	3.611832	0.891617
H	2.305859	3.209255	-0.900564
H	-0.089657	2.557309	0.258078
H	1.029715	1.795034	1.498000
H	4.290078	2.019654	1.141232
H	2.038908	-0.930324	-1.002754
H	3.736899	-0.690877	0.755494
H	2.637146	0.430127	1.707202
H	4.188822	1.614846	-0.650751

CCCC-adduct
E = −2065.26
N_{imag} = 0

C	0.689323	0.166199	0.146593
C	1.933662	2.285420	0.724050
C	0.577824	1.650468	0.381041
C	1.854645	-0.468231	-0.031365
C	3.187481	0.233961	-0.060865
C	3.036522	1.755990	-0.201285
H	-0.240524	-0.407660	0.129192
H	2.192214	2.041702	1.767154
H	1.867278	3.380894	0.662479
H	0.148649	2.131381	-0.516458

H	-0.147760	1.842575	1.187308
H	3.993323	2.254324	0.009581
H	1.858244	-1.552303	-0.169487
H	3.796517	-0.166175	-0.886987
H	3.749631	-0.009446	0.858754
H	2.776483	1.997534	-1.244548

CNCC - 2-azabutadiene
E = −1188.39
N_{imag} = 0

N	-2.365408	-0.841325	-0.893298
C	-1.880180	-1.546704	0.048243
C	-2.855533	0.441770	-0.636551
C	-2.161509	1.432966	-0.057780
H	-1.888334	-1.222139	1.103233
H	-1.439546	-2.520415	-0.188863
H	-3.848395	0.623436	-1.056633
H	-1.138297	1.283135	0.285826
H	-2.585990	2.431206	0.024832

TS-CNCC
E = −1903.46
N_{imag} = 1, ν = −390.275i cm^{-1}

N	-1.009335	-0.773157	-0.724736
C	1.701170	-0.538983	0.103158
C	-0.368209	-1.382660	0.248950
C	-1.213263	0.565413	-0.636178
C	-0.705889	1.385263	0.344413
C	1.607539	0.834942	0.103870
H	1.803403	1.406537	1.009264
H	1.845243	-1.074214	-0.832557
H	2.004512	-1.065105	1.007818
H	-0.392861	-1.036933	1.292650
H	-0.129361	-2.439722	0.108239
H	1.633547	1.396006	-0.826569
H	-1.683735	1.012497	-1.516652
H	-0.472465	1.017042	1.339336
H	-0.804626	2.465912	0.248982

CNCC-adduct
E = −1959.53
N_{imag} = 0

N	0.285711	-0.542353	0.107204
C	1.581558	1.538836	0.784633
C	0.275455	0.914492	0.280929
C	1.404711	-1.151818	0.064513
C	2.790872	-0.550619	0.140586
C	2.769291	0.975037	-0.001408
H	2.660526	1.241901	-1.064276
H	1.707466	1.306608	1.854357
H	1.530073	2.633771	0.700332

H	-0.010041	1.347427	-0.693212
H	-0.556221	1.148393	0.961941
H	3.718693	1.410222	0.338669
H	1.359612	-2.245485	-0.047957
H	3.419250	-1.021401	-0.631499
H	3.240261	-0.850465	1.103951

CNNC - 2,3-diazabutadiene
E = –1074.04
N_{imag} = 0

C	-0.729122	1.154284	0.767083
N	-1.346616	0.123555	1.202845
N	-1.577059	-0.925657	0.394146
C	-0.781869	-1.926297	0.388522
H	-0.626444	2.003988	1.442086
H	-0.319298	1.218228	-0.249776
H	0.135997	-1.961703	0.990381
H	-1.048580	-2.779637	-0.235074

TS-CNNC
E = –1779.02
N_{imag} = 1, ν = –373.558i cm^{-1}

C	-0.408213	1.346001	-0.473510
N	-1.145978	0.739323	0.427553
N	-1.206607	-0.625452	0.436338
C	-0.526311	-1.306647	-0.456645
C	1.631248	-0.757259	0.117482
C	1.692301	0.615417	0.108858
H	-0.324510	2.429064	-0.365624
H	-0.152801	0.936781	-1.458731
H	-0.236393	-0.934422	-1.447022
H	-0.538718	-2.391376	-0.334735
H	2.008089	-1.333936	-0.726359
H	1.502723	-1.298159	1.051987
H	2.118472	1.145736	-0.741885
H	1.612831	1.177267	1.036413

CNNC-adduct
E = –1832.41
N_{imag} = 0

N	-0.530407	0.322459	-0.310382
C	-0.764643	0.028294	2.234751
C	-1.220019	0.722649	0.958329
N	0.530407	-0.322459	-0.310382
C	1.220019	-0.722649	0.958329
C	0.764643	-0.028294	2.234751
H	-1.172982	-0.993508	2.269219
H	-1.152726	0.557505	3.115870
H	-1.082913	1.815241	1.016498
H	-2.287011	0.564565	0.749984
H	2.287011	-0.564565	0.749984

H	1.082913	-1.815241	1.016498
H	1.172982	0.993508	2.269219
H	1.152726	-0.557505	3.115870

NCCC - 1-azabutadiene
E = −1193.85
N_{imag} = 0

C	-1.536702	-1.400346	-0.227488
C	-2.784116	-0.912551	-0.219332
C	-3.112611	0.524505	-0.188889
N	-2.214588	1.438305	-0.163585
H	-1.348018	-2.472555	-0.250243
H	-0.679230	-0.727420	-0.211474
H	-3.637176	-1.594763	-0.235887
H	-4.193431	0.751547	-0.189293
H	-2.646242	2.370062	-0.145051

TS-NCCC
E = −1907.70
N_{imag} = 1, ν = −421.836i cm^{-1}

C	-0.220156	-1.433338	-0.503783
C	-1.220462	-0.865133	0.265574
C	-1.365454	0.540475	0.272818
N	-0.542763	1.275501	-0.450654
H	-0.020293	-2.502773	-0.441960
H	0.047162	-0.941384	-1.436238
H	-1.747717	-1.449318	1.020603
H	-2.079837	0.988099	0.978826
H	-0.658300	2.274336	-0.243329
C	1.545161	0.780476	0.114442
C	1.655918	-0.590844	0.260403
H	1.412303	1.420103	0.983636
H	1.908251	1.270200	-0.785360
H	1.586136	-1.040890	1.248451
H	2.213915	-1.164556	-0.478231

NCCC-adduct
E = −1958.49
N_{imag} = 0

N	-1.278202	-0.120006	-0.141630
C	-0.042866	1.885271	0.404245
C	-1.199351	1.207447	0.263492
C	-0.028352	-0.847255	-0.374536
C	1.117696	-0.323807	0.503286
C	1.291046	1.190542	0.302247
H	-2.047686	-0.656628	0.241783
H	1.981631	1.588811	1.061792
H	-0.079774	2.948336	0.635633
H	-2.165127	1.691629	0.416818
H	1.774239	1.386251	-0.671350
H	0.253504	-0.737180	-1.435804

H	-0.206415	-1.915977	-0.198622
H	2.043575	-0.863745	0.261597
H	0.883511	-0.526198	1.559386

OCCC - 1-oxabutadiene
E = −1078.62
N_{imag} = 0

C	3.818550	-4.173033	0.000000
C	2.634131	-3.548699	0.000000
C	1.348641	-4.292309	0.000000
O	1.248470	-5.507840	0.000000
H	3.860319	-5.263037	0.000000
H	4.758428	-3.623082	0.000000
H	2.567878	-2.458195	0.000000
H	0.433329	-3.650462	0.000000

TS-OCCC
E = −1790.61
N_{imag} = 1, ν = −427.797i cm^{-1}

C	-0.497414	1.310551	-0.425543
C	-1.315866	0.505250	0.369602
C	-1.086279	-0.886124	0.341524
O	-0.171157	-1.392910	-0.369404
H	-0.237548	0.930141	-1.411646
H	-0.579950	2.395161	-0.348441
H	-1.935190	0.928536	1.161443
H	-1.639698	-1.534163	1.051373
H	1.813996	1.753219	-0.559828
H	1.363432	1.434886	1.192613
C	1.736658	-0.301593	0.000254
C	1.446952	1.041717	0.180432
H	1.806643	-0.990072	0.836693
H	2.098935	-0.673806	-0.954683

OCCC-adduct
E = −1834.44
N_{imag} = 0

O	-1.302492	0.354682	-0.302999
C	0.011310	2.201981	0.665432
C	-1.220807	1.787408	-0.132321
C	-0.112868	-0.237842	-0.634142
C	1.092059	0.348595	-0.588995
C	1.283076	1.747440	-0.065963
H	2.144817	1.780111	0.618249
H	-0.037541	1.733684	1.659388
H	-0.001218	3.290613	0.815135
H	-1.201023	2.247497	-1.135221
H	-2.154149	2.071561	0.367152
H	-0.265776	-1.272521	-0.940144
H	1.956282	-0.235849	-0.901778
H	1.525161	2.445025	-0.885871

NCCN - 1,4-diazabutadiene

E = −1086.99

$\boldsymbol{N}_{\mathbf{imag}}$ = 0

C	1.974339	-0.756614	0.168395
N	0.945287	-1.459867	-0.103559
N	0.991970	1.433907	-0.177645
C	2.037704	0.705739	-0.117238
H	2.903283	-1.168548	0.604703
H	1.099268	-2.440936	0.166618
H	1.245673	2.410076	-0.382382
H	3.065872	1.094183	-0.239729

TS-NCCN

E = −1799.67

$\boldsymbol{N}_{\mathbf{imag}}$ = 1, ν = −406.794i cm^{-1}

C	-1.656096	-0.647872	-0.181368
C	1.187973	-0.745623	0.297681
N	0.323909	-1.364906	-0.484523
C	-1.612325	0.739576	-0.202834
N	0.408423	1.321061	-0.525459
C	1.232737	0.672776	0.275960
H	-1.745579	-1.171720	0.768404
H	-2.000988	-1.188840	-1.058323
H	1.753209	-1.259434	1.087805
H	0.254505	-2.358838	-0.229173
H	-1.668017	1.297068	0.730194
H	-1.923316	1.273792	-1.096463
H	0.401941	2.324705	-0.300590
H	1.830020	1.173803	1.050526

NCCN-adduct

E = −1848.42

$\boldsymbol{N}_{\mathbf{imag}}$ = 0

C	0.079324	0.469888	0.554125
C	1.810819	2.114430	0.313726
N	0.425077	1.880740	0.407832
C	0.799846	-0.311119	-0.561744
N	2.249508	-0.113048	-0.532053
C	2.675047	1.157376	-0.083343
H	-1.009183	0.350262	0.466135
H	0.397405	0.060281	1.532594
H	2.149922	3.127267	0.517360
H	-0.047690	2.490213	1.068691
H	0.412889	0.036929	-1.531519
H	0.579848	-1.382843	-0.480762
H	2.762587	-0.885750	-0.114602
H	3.738834	1.368098	-0.174508

OCCO - 1,4-dioxabutadiene

E = −850.83

N_{imag} = 0

C	0.000000	-0.776664	0.654451
C	0.000000	0.776664	0.654451
O	0.000000	1.435909	-0.356116
O	0.000000	-1.435909	-0.356116
H	0.000000	-1.239989	1.675163
H	0.000000	1.239989	1.675163

TS-OCCO

E = −1556.91

N_{imag} = 1, ν = −498.766i cm^{-1}

C	1.179498	0.693644	0.396719
C	1.163882	-0.718930	0.359820
O	0.246447	-1.329010	-0.286871
O	0.276032	1.356624	-0.216746
H	1.827423	1.201372	1.134255
H	1.800004	-1.278588	1.069556
H	-1.940593	-1.180637	-0.814066
H	-1.631553	-1.238304	0.997394
C	-1.541943	0.727072	0.105270
C	-1.557507	-0.677217	0.069118
H	-1.604518	1.241156	1.061197
H	-1.913024	1.283544	-0.750795

OCCO-adduct

E = −1598.87

N_{imag} = 0

C	-0.634967	0.216922	0.008336
C	-0.747682	-0.147140	2.304478
O	-1.375967	0.435067	1.151521
C	0.634967	-0.216922	0.008336
O	1.375967	-0.435067	1.151521
C	0.747682	0.147140	2.304478
H	-1.182084	0.426165	-0.906601
H	-0.913162	-1.236946	2.295391
H	-1.238300	0.288218	3.183320
H	1.182084	-0.426165	-0.906601
H	0.913162	1.236946	2.295391
H	1.238300	-0.288218	3.183320

NNCC - 1,2-diazabutadiene

E = −1067.35

N_{imag} = 0

N	-2.804581	-0.324887	-0.343715
N	-2.264390	-1.144366	0.438275
C	-2.364194	1.015202	-0.212065
C	-1.082859	1.370041	-0.060136
H	-0.786832	2.417014	-0.077113
H	-2.630724	-2.080090	0.168844
H	-3.155935	1.742959	-0.395126
H	-0.306976	0.612506	0.041455

TS-NNCC
E = −1787.29
N_{imag} = 1, ν = −317.557i cm^{-1}

N	-1.219996	-0.522991	-0.492720
C	1.661570	-0.859547	0.067936
N	-0.557738	-1.141737	0.426801
C	-1.043996	0.839721	-0.487496
C	-0.177964	1.488571	0.352202
C	1.895158	0.464581	-0.195962
H	1.890538	0.841792	-1.216138
H	1.531399	-1.570288	-0.745340
H	1.874959	-1.279632	1.048057
H	0.019749	2.551581	0.224631
H	-0.659614	-2.150473	0.218928
H	-1.537081	1.352207	-1.313930
H	2.347502	1.110955	0.554128
H	0.083037	1.029460	1.301230

NNCC-adduct
E = −1843.00
N_{imag} = 0

N	2.371880	0.529533	-1.042495
C	3.509640	2.012662	0.520479
N	2.431540	1.764178	-0.436041
C	3.457611	-0.156690	-1.144300
C	4.828276	0.249610	-0.658489
C	4.841964	1.704614	-0.164747
H	5.678718	1.881658	0.523403
H	3.400876	1.374408	1.419700
H	3.450902	3.062096	0.837885
H	5.147120	-0.440997	0.140436
H	1.503166	2.020593	-0.113315
H	3.351845	-1.109948	-1.668678
H	4.964044	2.389153	-1.015402
H	5.555420	0.109462	-1.473770

NCNC - 1,3-diazabutadiene
E = −1090.38
N_{imag} = 0

N	-1.765599	-0.710723	0.601395
C	-1.340830	-1.463350	-0.331482
N	-0.877662	1.367726	-0.017681
C	-1.865895	0.668199	0.391651
H	-1.131958	2.362834	-0.033948
H	-1.103519	-1.085521	-1.338436
H	-1.205089	-2.531316	-0.132254
H	-2.845761	1.067792	0.698789

TS-NCNC
E = −1807.19

N_{imag} = 1, ν = −368.881i cm^{-1}

C	1.744126	-0.646280	-0.099243
N	-1.045847	-0.743472	0.601732
C	-0.321379	-1.349708	-0.318529
C	1.658635	0.711857	-0.291910
N	-0.550909	1.281199	-0.446907
C	-1.212030	0.597478	0.450350
H	1.856308	-1.052233	0.904115
H	-0.675426	2.290686	-0.309151
H	2.061695	-1.289233	-0.919273
H	-0.299532	-0.978577	-1.351380
H	-0.088255	-2.404212	-0.154884
H	1.805017	1.147714	-1.276480
H	1.699068	1.400210	0.548694
H	-1.867605	1.067507	1.196773

NCNC-adduct

E = −1859.79

N_{imag} = 0

C	-1.098884	-0.708652	0.289789
N	0.069006	1.493738	-0.007503
C	-1.210732	0.782138	-0.062627
C	-0.033781	-1.372020	-0.588569
N	1.168467	-0.542387	-0.585949
C	1.115237	0.789584	-0.248217
H	-0.810169	-0.805994	1.346746
H	2.076077	-0.984420	-0.652521
H	-2.062765	-1.219360	0.161086
H	-1.640671	0.898122	-1.073385
H	-1.908202	1.282140	0.624652
H	-0.412767	-1.492220	-1.617531
H	0.217850	-2.373966	-0.213856
H	2.099059	1.271762	-0.202354
